# Real-Time Measurement of F-Actin Remodelling during Exocytosis Using Lifeact-EGFP Transgenic Animals

**DOI:** 10.1371/journal.pone.0039815

**Published:** 2012-07-02

**Authors:** Yujin Jang, Carolina Soekmadji, Justin M. Mitchell, Walter G. Thomas, Peter Thorn

**Affiliations:** 1 School of Biomedical Sciences, The University of Queensland, St Lucia, Queensland, Australia; 2 The Australian Prostate Research Centre – Queensland, Princess Alexandra Hospital, Buranda, Queensland, Australia; Université de Genève, Switzerland

## Abstract

F-actin remodelling is essential for a wide variety of cell processes. It is important in exocytosis, where F-actin coats fusing exocytic granules. The purpose of these F-actin coats is unknown. They may be important in stabilizing the fused granules, they may play a contractile role and promote expulsion of granule content and finally may be important in endocytosis. To elucidate these functions of F-actin remodelling requires a reliable method to visualize F-actin dynamics in living cells. The recent development of Lifeact-EGFP transgenic animals offers such an opportunity. Here, we studied the characteristics of exocytosis in pancreatic acinar cells obtained from the Lifeact-EGFP transgenic mice. We show that the time-course of agonist-evoked exocytic events and the kinetics of each single exocytic event are the same for wild type and Lifeact-EGFP transgenic animals. We conclude that Lifeact-EGFP animals are a good model to study of exocytosis and reveal that F-actin coating is dependent on the *de novo* synthesis of F-actin and that development of actin polymerization occurs simultaneously in all regions of the granule. Our insights using the Lifeact-EGFP mice demonstrate that F-actin coating occurs after granule fusion and is a granule-wide event.

## Introduction

F-actin remodelling has long been associated with the process of exocytosis [1]. In some cell types, such as acinar cells, oocytes, endothelial cells and alveolar cells, this remodelling is observed as an F-actin coating of the fusing exocytic granules [2], [3], [4], [5], [6], [7]. This F-actin coating is likely to take place after fusion [5] and probably requires the genesis of new F-actin filaments with evidence for nucleation by the small G-proteins, Cdc-42 [4] and Rho [6]. Most of the data for F-actin coating comes from fixed-cell studies and phalloidin staining [5], [6]. Injected Alexa488-labelled-G-actin has also been used to track the coating in real time and it decorates the fused granule over a period of a few seconds [4]. Further, a recent paper has shown F-actin coating in salivary acinar cells using viral infection of the new probe Lifeact-EGFP [8], which has a low-affinity interaction with F-actin [9]. These latter two live-cell studies have significantly enhanced our understanding of the involvement of F-actin in exocytosis but the problems with microinjection in the former [4] and viral infection in the latter [9] limit their use. There are also well documented problems with G-actin-based fluorescent protein probes [10] interfering with F-actin dynamics [11]. The production of the Lifeact-EGFP transgenic mouse now opens up potential new opportunities to study actin remodelling, in all cells, within native tissues [12]. Critical to this use though is the validation of Lifeact-EGFP as a reporter that doesn't interfere with physiological processes [10].

We have tested the use of Lifeact-EGFP transgenic mice using a sensitive assay for exocytosis where we can record the time-course of each individual fusion event and the single-granule kinetics of fusion [13], [14]. Our results compare responses in the exocrine pancreas of wild type mice with transgenic Lifeact-EGFP mice and show no differences either in the time course of agonist-induced exocytic events or in the kinetics of each single fusion event. Using the Lifeact-EGFP pancreas, we now reveal that F-actin coating occurs rapidly after granule fusion. We show that latrunculin prevents the F-actin coating indicating it is due to actin nucleation and not movement of F-actin. Finally, we show that this F-actin coating develops simultaneously across all regions of the granule. We conclude that the Lifeact-EGFP animals are a useful tool to study F-actin remodelling during exocytosis.

## Results

**Figure 1:**
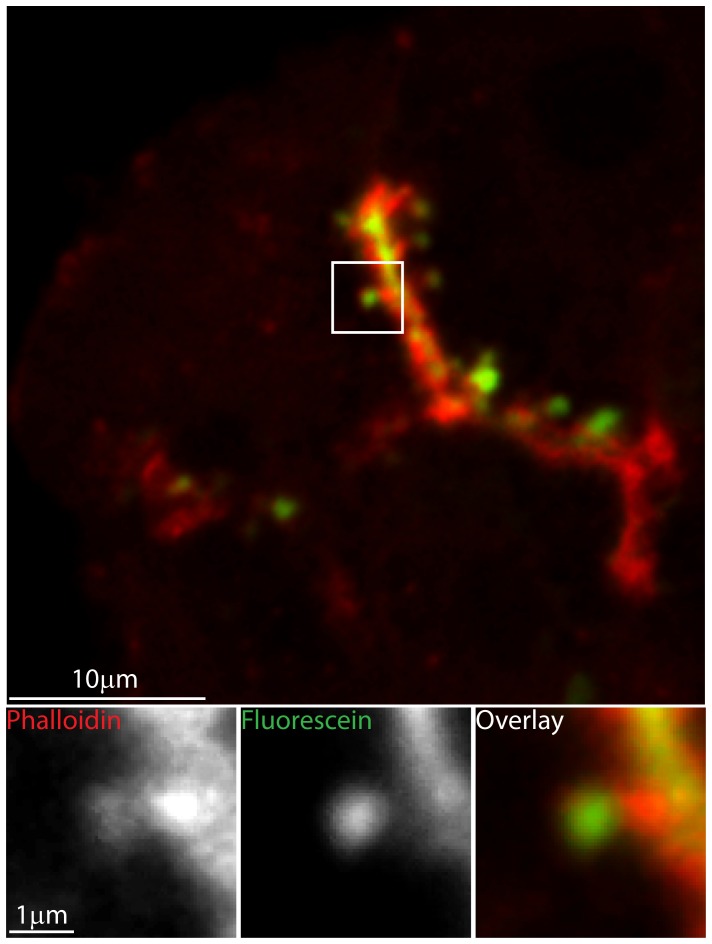
F-actin coats individual fused granules. Pancreatic tissue fragments were bathed in paraformaldehyde-fixable fluorescein extracellular dye and then stimulated with 1 µM acetylcholine. Each fused granule is then identified by the uptake of fluorescent dye. After fixation, co-staining with phalloidin Alexa-633, shows that each fused granule is coated with F-actin. The upper panel shows a low magnification image, where-as the lower panel shows high magnification images that are enlargements of the boxed region.

In many cell types, F-actin remodelling during exocytosis appears as a coating of individual granules. Figure 1 shows the F-actin coating of fused granules in pancreatic acinar cells. For this, we bathe pancreatic fragments from wild type animals in lysine-fixable fluorescein dye (green), stimulate the cells with acetylcholine and then fix in paraformaldehyde. Upon fusion of the zymogen granules the extracellular fluorescein enters and therefore labels each individual fused granule. Counter-staining with phalloidin-Alexa 633 (red) labels the F-actin cytoskeleton. In this example, the low power image shows extracellular dye staining outlining the cells and in the lumen that lies between the cells, phalloidin stains the F-actin enriched in sub-apical regions. The enlarged images show a single fused granule filled with extracellular dye (green) and surrounded with an F-actin coat (red). These fixed-cell experiments give no insight into the timing and kinetics of F-actin coating, for this we require a live-cell stain for F-actin.

**Figure 2:**
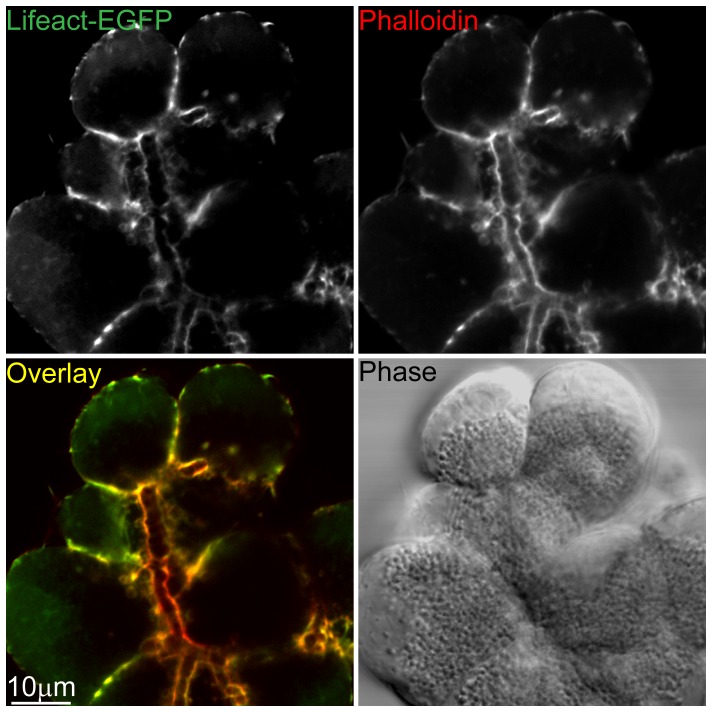
Lifeact-EGFP transgenic animals label F-actin in native mouse pancreatic acinar cells. Pancreatic fragments retain the secondary structure of the native gland. In this example a branching lumen runs top to bottom. It is surrounded by acinar cells and is the region against which zymogen granules are clustered (seen in the phase image). Lifeact-EGFP fluorescence (green) is enriched beneath the cell membrane particularly along the lumen, on the lateral region between the cells and in patches under the basal plasma membrane. This pattern of Lifeact-EGFP enrichment is very similar to the phalloidin (red) fluorescence. The differences in intensity of Lifeact-EGFP and phalloidin may be due to different pools of F-actin since it is known that the apical F-actin network is stable and partially resistant to latrunculin action [26].

A preparation of mouse pancreatic fragments from the Lifeact-EGFP transgenic animals shows strong fluorescent staining along the apical lumens and sub-cortical regions beneath the basolateral membranes. In paraformaldehyde-fixed cells, this Lifeact-EGFP fluorescence pattern was the same as that observed when phalloidin-Alexa 633 used as a counter-stain (Figure 2).

Both patterns of fluorescence are as previously described for F-actin distribution in resting exocrine pancreatic tissue [3] and indicate that Lifeact-EGFP faithfully labels F-actin. A particular advantage of using the Lifeact-EGFP animals compared to cell infection/transfection approaches is that all cells express the probe. However, for these animals to be experimentally useful it has to be demonstrated that this exogenous probe has minimal impact on F-actin remodelling and on the processes of interest; in our case regulated exocytosis.

**Figure 3:**
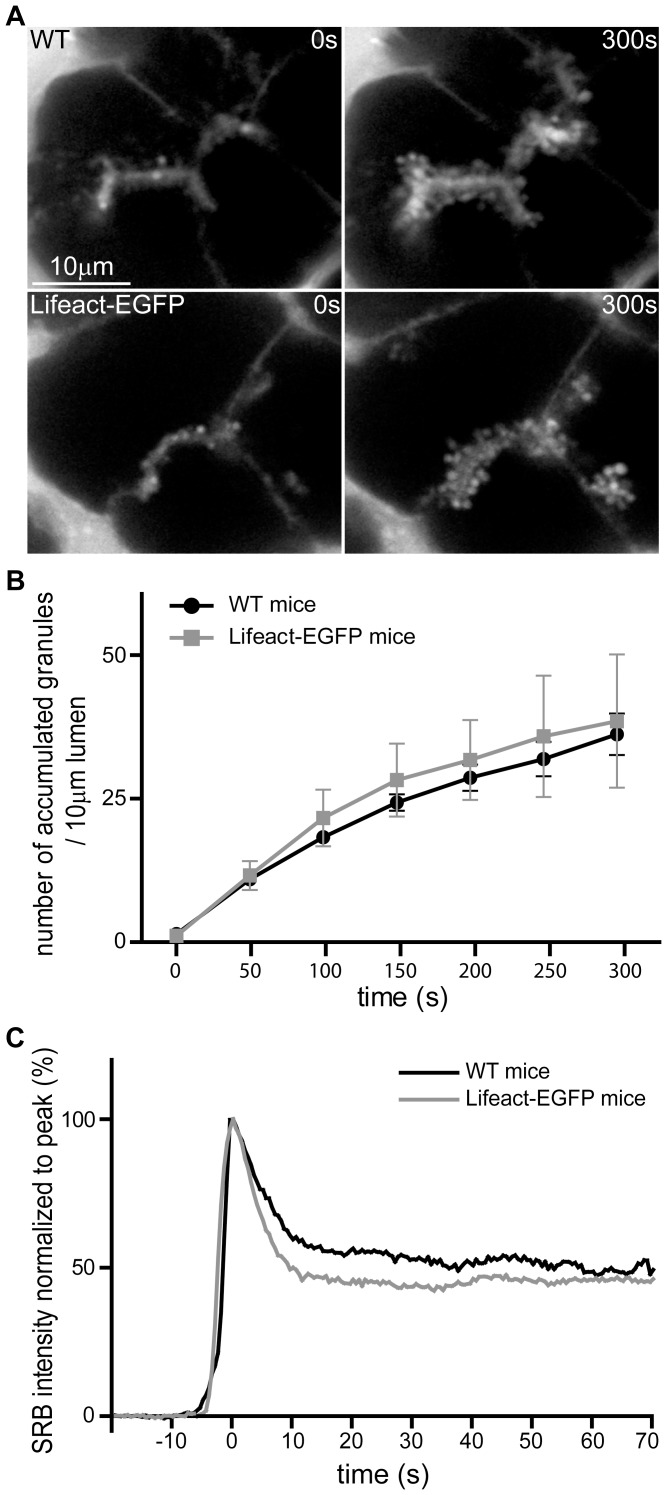
Lifeact-EGFP transgenic mice show similar acetylcholine-induced exocytic responses to wild type. (**A**) The images were taken before (0 s) and 300 s after exocytic fusion induced by 1 µM acetylcholine stimulation of mouse pancreatic fragments in wild type (upper images) and Lifeact-EGFP transgenic animals (lower images). The tissue fragments were bathed in extracellular fluorescent dye (SRB) which labels the lumens (bright fluorescence between the cells in control) and enters and labels each individual granule (bright fluorescent spots along the lumen after 300 s). (**B**) We identify the time point of appearance of each fusing granule (cumulative histogram, aligned to the first exocytic event) which shows both wild type and transgenic animals have a similar time-course and extent of exocytic response. (C) Further, we compare the fluorescence profile, over time, of SRB dye entry into each individual granule and observe no differences between wild type and transgenic animals.

To identify and quantify exocytosis in pancreatic acinar cells, we track, in living cells, the entry of extracellular fluorescent dye into each fused granule using two photon microscopy [13]. The two photon optical slice, in our machine, is ∼1 µm deep [13]. Within this depth we determine the time-course of exocytic fusion events in response to cell stimulation in control animals and in Lifeact-EGFP transgenic animals. In both cases, the response to 1 µM acetylcholine induces a similar time-course of fusion events (Figure 3, after 300 s, control cells have 35.6+/−3.5 events per 10 µm lumen (mean +/−SEM, n = 3 mice and 3–4 lumens per mouse) and cells from Lifeact-EGFP transgenic animals have 37.9+/−11.4 events per 10 µm lumen (mean +/−SEM, n = 3mice and 3–4 lumens per mouse) not significantly different Student's T test). Since exocytosis in this preparation has been shown to be affected by manipulation of F-actin [6], [15], [16] the similarity of the responses in Lifeact-EGFP transgenic and in wild type animals suggests the Lifeact-EGFP probe is not interfering with F-actin dynamics.

We also use the same method of dye entry into granules to track fluorescence changes within single granules after fusion [17]. These typically show a rapid peak of fluorescence after fusion and then a long-lasting plateau which reflects entry of dye into the granule, then binding of the dye to granule content (peak) and then loss of content leading to an empty “ghost” granule (plateau) [17]. The granule fluorescence is therefore an indirect marker of granule behaviour. Manipulation of F-actin is known to change granule behaviour leading to granule instability [6] and closure of the fusion pore [18]. In the experiments here single-granule fluorescence changes in both the wild type and Lifeact-EGFP follow a very similar peak-plateau profile (Figure 3, lower graph) which provides further confirmation that Lifeact-EGFP is not interfering with F-actin dynamics.

**Figure 4:**
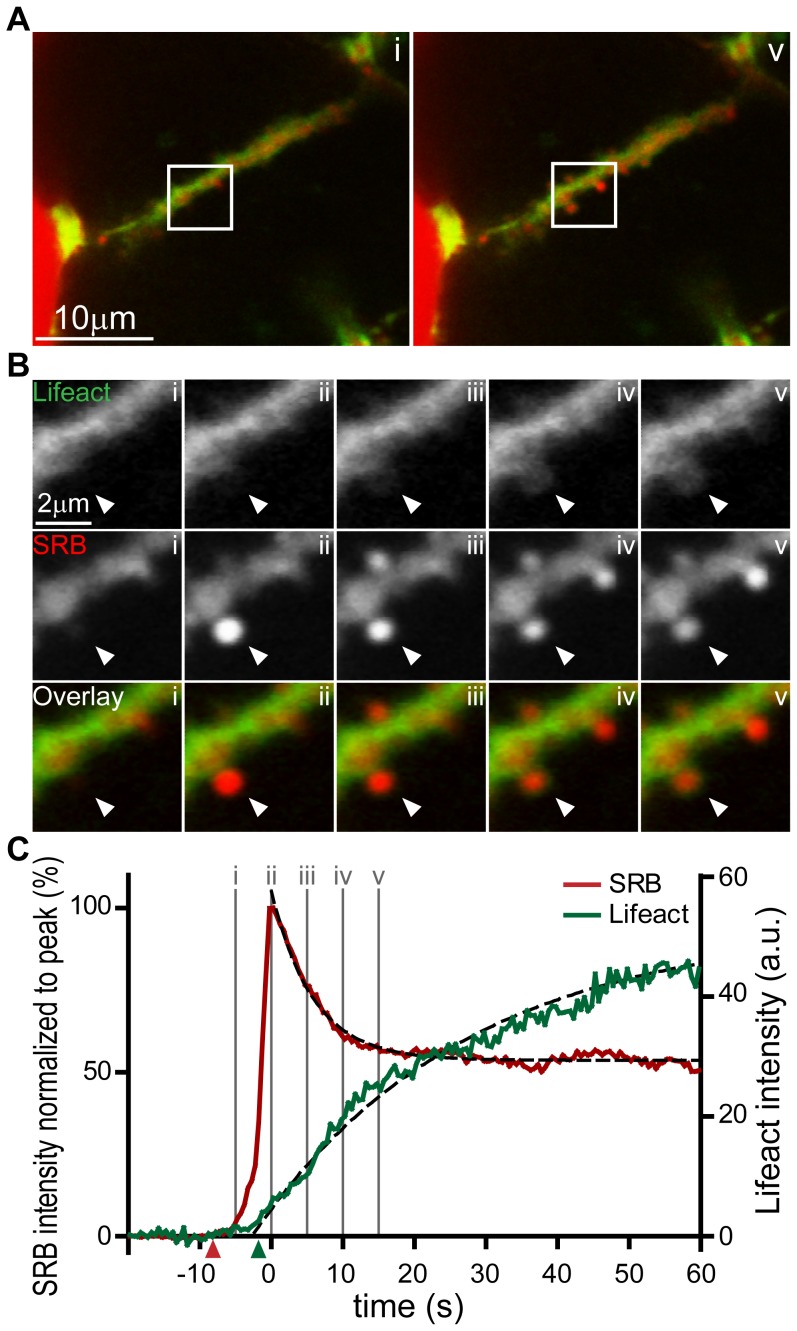
Real-time imaging of exocytic fusion events and F-actin coating. (**A**) Low magnification images show a lumen lying diagonally between two acinar cells identified with SRB (red) and Lifeact-EGFP (green) in the sub-apical region. (**A, i**) is an image taken before the appearance of exocytic events at the time point indicated “i” on the graph of fluorescence intensity over time in panel (**C**). (**A, v**) Is an image at a time point after induction of a number of exocytic events which can be seen as bright spots of SRB fluorescence along the lumen; the time point “v” is indicated on the lower graph (**C**). (**B**) Shows an image sequence from an enlarged region (box shown in **A**) of Lifeact-EGFP and SRB and the overlay, for two exocytic events. The images were taken at the time points i, ii, iii, iv, and v as indicated on the graph in panel (**C**). (**C**) is a graph of fluorescence changes over time taken from a region of interest placed over the lower exocytic event (indicated by an arrowhead). SRB fluorescence is plotted normalized to the peak and rises rapidly to a peak and then decays to a plateau. The simultaneously recorded Lifeact-EGFP signal, plotted in arbitrary fluorescence units, rises slowly and nearly reaches a maximum by the end of the record. The starting points of the SRB and Lifeact-EGFP signals, as determined by a positive deflection of the signal by more than 5 times the standard deviation of the signal noise, are shown by the colour-coded triangles on the X axis. The black dotted lines were mono-exponential fits to the data with τ values of 6.9 s (SRB) and 29.4 s (Lifeact-EGFP).

We conclude that the Lifeact-EGFP transgenic animals are a useful tool for the live study of F-actin remodelling during exocytosis. In acinar cells, it is clear that F-actin coating is specifically associated with fusing granules but the time-course of coating relative to fusion is not known. To answer this question we used isolated pancreatic fragments from Lifeact-EGFP mice bathed in extracellular solution containing the fluorescent dye sulforhodamine B (SRB) and imaged during an acetylcholine-induced response We simultaneously recorded the Lifeact-EGFP signal and extracellular dye (SRB) entry into single fusing granules, with entry of the dye as an index of the time-point of granule fusion. Single fusion events in response to 1 µM acetycholine stimulation were detected as the entry of extracellular SRB dye into the granule (Figure 4). From this time-point of fusion we observed a short delay of a few seconds (6.7+/−0.6 s, mean +/− SEM, n = 43 fusion events, Figure 4C triangles on graph indicate the times when the signal reached 5× the standard deviation of the noise) before the development of Lifeact-EGFP enrichment around each granule. This F-actin coating increased with a time-course that could be approximated by a single exponential (Figure 4). The calculated average fitted time constant for all fusion events studied was 27.9+/−4.1 seconds (n = 43 granules, mean +/− SEM).

**Figure 5:**
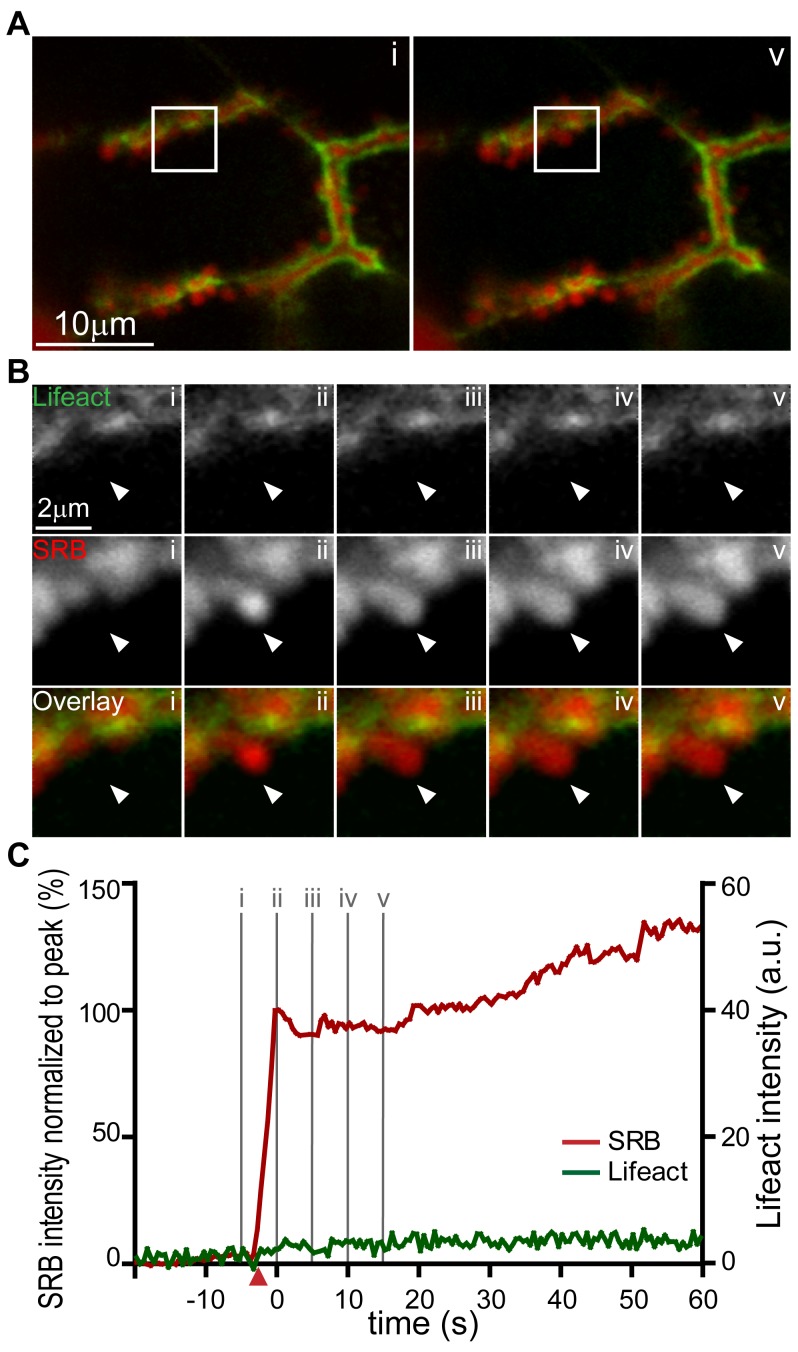
Latrunculin treatment abolishes F-actin coating of fused granules. (**A**) Low magnification images show complex lumens, identified by SRB (red) and Lifeact-EGFP fluorescence (green), lying between the cells within a pancreatic fragment. (**Ai**) is an image taken before the appearance of exocytic events, (**Av**) is taken after, at the time points “i” and “v” as indicated on the graph in panel (**C**). (**B**) Shows an image sequence from an enlarged region (box shown in A) of Lifeact-EGFP and SRB and the overlay, for a single exocytic event. The images were taken at the time points i, ii, iii, iv, and v as indicated on the graph in panel (**C**). (**C**) Is a graph of fluorescence changes over time taken from a region of interest placed over the exocytic event (indicated by an arrow). SRB fluorescence is plotted normalized to the first, rapid peak and shows a rapid rise followed by a slower increase. The simultaneously recorded Lifeact-EGFP signal, plotted as arbitrary fluorescence units, shows only very small changes over time.

Previous work has indicated that F-actin coating of the granule is dependent on actin polymerisation, rather than movement of F-actin around the granule [4], [6], [19]. To test this in live cells, we treated the cells for 10 minutes with 10 µM Latrunculin B, which sequesters G-actin monomers, and then stimulated with acetylcholine in the continued presence of latrunculin. This latrunculin treatment did not affect granule fusion and we still observed SRB entry into each fused granule (Figure 5). However, after latrunculin treatment the Lifeact-EGFP signal increased by only 1.43+/−0.17 (mean +/− SEM, n = 50) arbitrary fluorescent units when comparing the fluoresecent signal 10 s before the exocytic event with the signal 50 s after the event. This shows that latrunculin blocked changes to the Lifeact-EGFP signal around fused granules indicating that F-actin coating had been abolished (Figure 5, n = 50 fusion events). In the case of latrunculin treatment, we often observed merging, multiple fusion events. This activity affects the SRB signal and often led to progressive increases in SRB fluorescence as shown in Figure 5. Notwithstanding this fusion behaviour we did not see the development of F-actin coats at any point in the responses.

**Figure 6:**
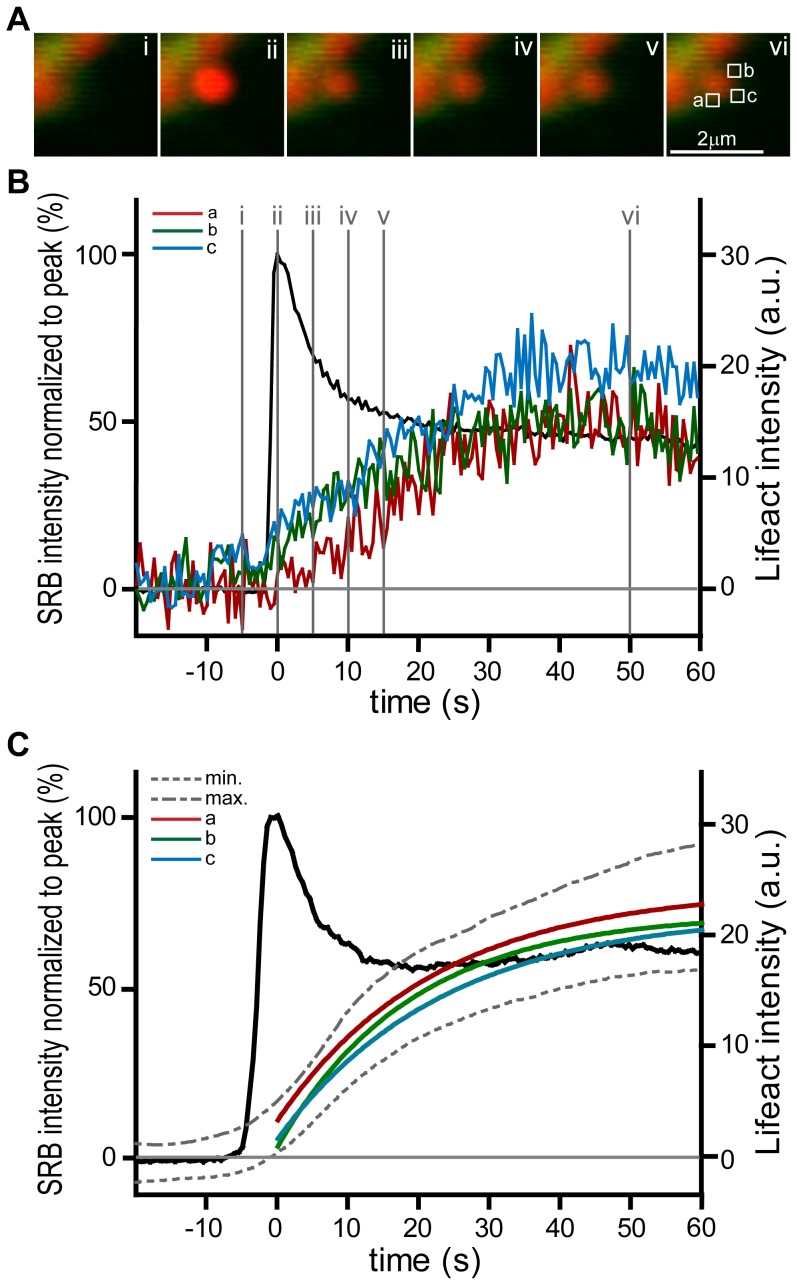
F-actin coating is initiated simultaneously across the whole of the granule. (A) a high magnification image sequence (i, ii, iii, iv, v with times shown on graph in **B**) shows SRB entry and Lifeact-EGFP tracking of F-actin coating of an fusing individual granule. (**B**) Regions of interest (boxes a, b, c shown in **A**) placed around single fusing granules showed only small temporal differences in the time-course of F-actin coating. When fitted to a single exponential the τ values were 15.5, 13.3 and 15.5 seconds (for regions a, b, c respectively). (**C**) shows the graph obtained from the means of 10 events. The mean SRB signal from each granule is shown for reference (black plot). For each event regions a and b were placed at the luminal-granule interface and c was placed at the top of the granule. The extreme minima (labelled min) and maxima (labelled max) of the SEM from all 3 regions of interest are shown as dotted grey lines. The average Lifeact-EGFP change within each region was fitted to a single exponential and these fitted curves are shown, colour-coded, on the graph.

We conclude that F-actin coating occurs after fusion and is dependent on actin polymerization. Small G-proteins have been implicated in F-actin coating and it has been proposed that their activation is dependent on the mixing of components from the plasma membrane and the granule membrane [4], [20]. We can now shed some light on the process of F-actin polymerisation by recording the spatial dynamics of Lifeact-EGFP by placing small regions of interest around the granule (Figure 6, n = 10 granules). This shows that, within the limitation of our resolution, F-actin polymerization initiates simultaneously in each of the regions, across the whole of the granule membrane (Figure 5B). While we cannot rule out that initiation might start at the luminal-granule interface and spread rapidly across the granule, our data does show that after initiation, the F-actin density increases relatively slowly over time. This slow increase approximates to a single exponential with similar fitted mean time constants for each region of 22.4+/−2.7, 18.8+/−2.3 and 23.5+/−2.0 seconds (for regions a, b, c respectively, mean +/− SEM) as shown in Figure 6C.

## Discussion

Here we demonstrate that pancreatic acinar cells taken from Lifeact-EGFP transgenic animals show no differences in exocytosis measured in terms of the numbers of fusion events induced by agonist and in terms of the kinetics of fusion of single granules. Since it is well known, in these cells, that pharmacological manipulation of the F-actin cytoskeleton does affect both these parameters [3], [6], [16], we conclude that the Lifeact-EGFP probe has little impact on F-actin dynamics during exocytosis. The fact that in transgenic animals it is expressed in every cell adds to the utility of this probe and ease of use in imaging experiments.

Using preparations of exocrine pancreatic fragments, we now directly demonstrate that F-actin remodelling occurs after granule fusion and is seen as a coating around each fused granule. Latrunculin inhibits this coating indicating that it is due to nucleation and polymerization of G-actin, rather than movement of F-actin. Finally, we show that development of F-actin polymerisation occurs with a similar time course across all regions of the granule.

These observations extend our knowledge of F-actin remodelling in acinar cells and provide new insights into the possible mechanisms. Firstly, the initial observations of F-actin coating of granules [3] showed an association with Rab3D dynamics but could not distinguish whether coating occurred before or after excoytic fusion. Work from our laboratory then showed that each F-actin coated granule was filled with an extracellular marker and that some granules contained the marker but not the F-actin coat [5]. This implied that F-actin coating follows fusion and was consistent with observations made in oocytes [4]. Our data now show that F-actin coating occurs with a lag of a few seconds after granule fusion. Secondly, in principle, the F-actin coat could occur either as a result of a reorganization of the existing cortical F-actin network or due to *de novo* synthesis of F-actin. Our data, with short-lasting treatment with latrunculin (10 minutes pre-treatment and continued presence during stimulation), which sequesters G-actin monomers, now shows complete block of coating. This indicates that coat dynamics require nucleation since short-lasting latrunculin treatment would not be expected to affect movement of existent F-actin filaments. This conclusion is consistent with previous observations in fixed cells [6]. Since G-actin is present on granule membranes [21] we speculate that granule fusion triggers polymerisation from this local actin pool.

Our method has a temporal resolution of 2 frames per second and within this limitation we do not observe any difference in the timing of F-actin formation across the granule (Figure 6). These latter observations are consistent with ideas derived from work in oocytes that post-fusion changes in the granule are the trigger for F-actin coating [4]. In oocytes it has been suggested that nucleation is initiated after the mixing of the granule membrane with the cell membrane and that Cdc42 is a component in this action. It is possible that separate components of nucleating machinery exist on the plasma membrane and granule membrane. Granule fusion might then bring these components together and start F-actin nucleation [22].

Direct comparison of our data with that of Yu and Bement in oocytes [20] is interesting. The granules in the Xenopus oocytes are ∼4.3 µm in diameter and show complete coating (using an Alexa 488-G actin probe) at around 17.5 s after fusion. Thereafter the coating increases in intensity with a time constant of around 20 s [20]. In acinar cells, the granules are about 1 µm in diameter which calculates to 18 fold less surface area than oocyte granules. From the oocyte work we would therefore predict complete coating of zymogen granules at 1 s. This is faster than our temporal resolution and so we cannot use our method to determine where the initiation of nucleation takes place. However the subsequent build-up of F-actin is slow (mean τ = 29.48 s). It occurs with the same time course across all regions of the granule (Figure 6) and is comparable to the time course seen in the much larger oocyte granules. This indicates that development of actin polymerisation is driven by a process that is independent of granule size. It also suggests that it follows a similar mechanism in both oocytes and acinar cells.

There is currently no good understanding of the potential role of F-actin coating. In the pancreas, it has been suggested that it might stabilize the granule [6] maintaining its shape over time. On the other hand, in other cell types, it has been suggested to play an active role is contracting around the granule and forcing expulsion of granule content [4], [7], [23]. Our data argue for a stabilization function. We know that content loss from the granule is approximately exponential, with a time constant of 6.1 s (Figure 4 and see [17]). Our new data, presented here, shows that the accumulation of F-actin is much slower than this, with a mean time constant of 27.9 seconds, and furthermore there is no evidence for a contraction or disappearance of the granule as in other cell types [23]. It therefore seems unlikely that the F-actin coat is important for squeezing out of content in the pancreas.

While F-actin coating may not play a role in expelling content, F-actin may play another role in the loss of content. The data of Figure 5 show that with latrunculin treatment the SRB signal remains elevated over time (as opposed to the peak-plateau seen in the control in Figure 4). We have previously shown the drop to a lower plateau is due to loss of content [17]. This suggests that the elevated SRB fluorescence with latrunculin treatment may be due to content retention. One possible explanation is that latrunculin treatment causes the closure of the fusion pore [16] and if this closure occurred soon after fusion then content would become trapped. If this is correct then the F-actin coat could be distinct from F-actin around the neck of the fusion pore and the two pools of F-actin may have separate functions.

If the F-actin coat's primary role in acinar cells is stabilization we are still left with the question as to what is the purpose of this stabilization. Possibilities include stabilization of the apical region of the acinar cell and the acinus structure. This region is heavily enriched with F-actin (see Figure 2) and in conjunction with adherens and tight junctions forms the multicellular, three dimensional structure of the acinus. So at rest the apical membrane is mechanically supported by the F-actin network and the insertion of granules into this membrane may require F-actin coating to maintain the structural integrity of the region. The second possibility is that the F-actin coat is engaged in post-fusion events such as providing a scaffold for endocytic protein complexes and/or providing support for secondary granule fusion during compound exocytosis [24].

In conclusion we provide a detailed analysis indicating that exocytosis in the exocrine pancreas is not affected by the presence of a Lifeact-EGFP low affinity F-actin probe. Our data show that F-actin remodelling occurs as a result of polymerisation triggered rapidly after granule fusion. The Lifeact-EGFP transgenic animals are likely to be a useful tool in unravelling the role actin plays during secretion in a wide range of cell types.

## Methods

### Cell preparation

Transgenic Lifeact-EGFP mice were obtained as a gift from Professor Roland Wedlich-Soldner [12]. These transgenic mice were produced on a 128SV/C57BL/6 background using a cytomegalovirus enhancer, chicken actin promoter to drive expression of Lifeact-GFP in all tissues [12]. Mice were humanely killed according to local animal ethics procedures and approved by the Anatomical Biosciences Ethics Committee of the University of Queensland (AEC SBMS/089/09/ARC (NF)). Isolated mouse pancreatic tissue was prepared by a collagenase digestion method in normal NaCl-rich extracellular solution [25], modified to reduce the time in collagenase and limit mechanical trituration. The resultant preparation was composed mainly of pancreatic lobules and fragments (50–100 cells), which were plated onto poly-L-lysine-coated glass coverslips and used within 3 hours of isolation from the animal.

### Experimental procedures

After preparation of the pancreatic fragments and adherence to the poly-l-lysine coated coverslips, we washed the cells in extracellular solution. Cell stimulation was performed by addition of a bolus of acetylcholine stock solution to the solution surrounding the cells. In the latrunculin experiments, the drug was applied 10 minutes before cell stimulation and was present throughout stimulation with acetylcholine.

### Confocal Imaging

Fixed specimens were imaged using an Olympus BX61 upright confocal laser scanning microscope, with a 63× objective lens (NA 1.3), and an optical slice depth of ∼1 µm. Images were collected with the appropriate filters and captured in sequential tracks to minimize crosstalk to less than 2%. Fluorescent probes were from Molecular Probes Inc. (Eugene, OR, USA). All other compounds were from Sigma (Sigma Aldrich, Australia). All experiments were performed at least three times. We used Metamorph software for analysis of LSM images and Adobe Photoshop for image processing and presentation.

### Indirect immunofluorescence

Cell preparations were fixed in freshly prepared 4% paraformaldehyde in PBS for 30 minutes, and permeabilzed with 0.5% Triton-X 100 in PBS for 10 minutes. F-actin was visualized with phalloidin Alexa 633 (Invitrogen, Molecular probes).

### Live-cell Two-photon Imaging

We used a custom-made, video-rate, 2-photon microscope using a Sapphire-Ti laser [13], with a 60× oil immersion objective (NA 1.42, Olympus), providing an axial resolution (full width, half maximum) of ∼1 µm. We imaged exocytic events using Sulforhodamine B (SRB, 20 µg.ml^−1^, Sigma) excited by femtosecond laser pulses at 850 nm, with fluorescence emission detected at 550–700 nm (SRB).

Images (resolution of 15 pixels/µm, average of 15 video frames) were analyzed with the Metamorph program (Molecular Devices Corporation). Exocytotic event kinetics were measured from regions of interest (0.78 µm^2^, 100 pixels) over granules. Traces were rejected if extensive movement was observed.

Data analysis was performed on the average data obtained from regions of interest placed over the granules. In all analyses we subtracted the background fluorescent signals. For analysis of the SRB recordings, we typically normalise all records to the amplitude of the first peak response. In the case of the Lifeact-EGFP signals these are expressed in arbitrary units.

To determine the time point of initiation of the signals in the experiments of Figure 4 we measured the noise in the fluorescent signal prior to the exocytic event. We then determined the time where the fluorescent signal increased by more than 5× the standard deviation of the noise away from the mean.

All data are shown as mean ± SEM and are obtained from at least three separate animals.

Images were recorded in TIF format and image panels produced within the Metamorph program.
